# The Gross Developmental Potential (GDP2): a new approach for measuring human potential and wellbeing

**DOI:** 10.1186/s12889-022-14030-x

**Published:** 2022-08-27

**Authors:** Neal Halfon, Anita Chandra, Jill S. Cannon, William Gardner, Christopher B. Forrest

**Affiliations:** 1 UCLA Center for Healthier Children Families, & Communities, Los Angeles, USA; 2 Department of Health Policy& Management, UCLA Fielding School of Public Health, Los Angeles, USA; 3 Department of Pediatrics, UCLA Geffen School of Medicine, Los Angeles, USA; 4 Department of Public Policy, School of Public Affairs, UCLA Luskin, Los Angeles, USA; 5 RAND Corporation, Arlington, USA; 6 RAND Corporation, Santa Monica, USA; 7 School of Epidemiology & Public Health, Department of Child & Adolescent Psychiatry, University of Ottawa, Ottawa, Canada; 8 Applied Clinical Research Center, Department of Pediatrics, Children’s Hospital of Philadelphia, University of Pennsylvania, Philadelphia, USA

**Keywords:** Wellbeing, Gross domestic product, Gross developmental potential, Human potential, Life-course health development, Adaption, Lifelong learning

## Abstract

Many factors influence the health and well-being of children and the adults they will become. Yet there are significant gaps in how trajectories of healthy development are measured, how the potential for leading a healthy life is evaluated, and how that information can guide upstream policies and investments. The Gross Developmental Potential (GDP2) is proposed as a new capabilities-based framework for assessing threats to thriving and understanding progress in achieving lifelong health and wellbeing. Moving beyond the Gross Domestic Product’s (GDP) focus on economic productivity as a measure of progress, the GDP2 focuses on seven essential developmental capabilities for lifelong health and wellbeing. The GDP2 capability domains include Health -living a healthy life; Needs-satisfying basic human requirements; Communication-expressing and understanding thoughts and feelings; Learning-lifelong learning; Adaption -adapting to change; Connections -connecting with others; and Community -engaging in the community. The project team utilized literature reviews and meetings with the subject and technical experts to develop the framework. The framework was then vetted in focus groups of community leaders from three diverse settings. The community leaders' input refined the domains and their applications. This prototype GDP2 framework will next be used to develop specific measures and indices and guide the development of community-level GDP2 dashboards for local sense-making, learning, and application.

## Background

The coronavirus disease 2019 (COVID-19) pandemic demonstrated how fractures in the U.S. health system and broader community systems could fail to assure the health and well-being of so many. Disproportionately affecting communities of color and exposing deep health inequities, this COVID 19 pandemic also exacted an unknown toll on the future health potential of many children whose lives were disrupted and developmental pathways marooned by school closures and other related adversities.

There are concerns now about social and emotional health impacts (e.g., school anxiety, depression) and learning loss, which will affect current youths' health and educational outcomes, and through them, the social and economic progress of the nation [1]. Unfortunately, our current data systems and social progress indicators do not provide the necessary metrics to assess these impacts and adapt to them in ways that will improve these trajectories.

Given the importance of assessing the development of health and well-being, where do we stand? There are significant gaps in how we track the development of children and youth, evaluate their potential, and predict how the pandemic will influence their future health, social, and economic prospects [2]. Too often, we use lagging well-being indicators (e.g., life expectancy, high school graduation, hospitalization rates) with less attention to upstream leading indicators that would enable us to intervene quickly and efficiently. Although lagging measures of economic output such as the Gross Domestic Product (GDP) are correlated with other measures of social progress, they do not directly measure the human capabilities necessary to achieve optimal levels of health and well-being. That GDP falls short as a measure of well-being is evidenced by the fact that the US – the large country with the highest GDP per capita – falls behind most of its OECD peers on life expectancy, subjective well-being, or educational attainment. Absent direct assessments of capability, we cannot monitor progress toward a society that supports the next generation’s creativity, innovation, and agility.

Many countries have sought to go beyond GDP as the primary marker of economic and social progress by using indices that provide a more holistic assessment of what it means to have a flourishing population. New Zealand, for example, focuses on a well-being economy and uses a range of social indicators to map national progress toward a well-being-promoting society [3]. Yet, there still appear to be sticking points in translating the science of human development into actionable guidance. Current alternative national measures have been less successful than GDP in aligning stakeholders and incentivizing them to act [4–6].

In a rapidly changing world, successful measurement strategies do not just document what is but also provide a direction for future social investments, policies, programs, and services. The best measurement systems turn data into information, and information into insights that inform new narratives, stimulate learning and become currency in the marketplace of social progress. Measuring the levels of human potential in our communities can inform upstream investments in developing essential capabilities. Making such investments during sensitive developmental periods can improve health and well-being outcomes. Not capturing this information impedes the capacity of economies and regions to thrive.

This paper reports on an effort to build a new framework for a new measure of social progress – gross developmental potential (GDP2) – that could complement the GDP. The GDP2 employs the innovative Developmental Capabilities Framework to describe those capabilities needed to thrive in the U.S. today and in the future [7]. There is no sentinel indicator of human potential for the nation that accounts for the drivers and influences that help individuals flourish over their lifespans. GDP2 attempts to capture the potential of a nation by assessing the developmental capabilities that promise to define their young people's life course trajectories, enabling them to thrive as youth and adults.

## Methods

The GDP2 development project sought to develop an approach for measuring the development of human potential in children and youth. The project analyzed a range of conceptual approaches for measuring human potential across the life course. Subject matter experts were recruited and interviewed iteratively as we created a conceptual framework and process for measuring the potential for well-being development. Once the initial set of developmental capabilities was identified, we solicited additional subject-matter expert input, conducted a focused literature review, and interviewed community leaders to refine our approach further.

### Subject matter experts

We convened two types of subject-matter experts: a) a technical expert panel (TEP) that met in person twice, with follow up teleconference discussions and reviews, and b) subject-matter expert key informant interviews to obtain input on the proposed overall GDP2 framework.

The TEP comprised 15 leaders in child health, developmental science, economics, psychology, community measurement, and social policy. The diverse group of individuals were selected not only for their technical measurement expertise but also for their experience working across sectors, levels of government, and in the social innovation space. The TEP’s first meeting addressed challenges and opportunities in measuring human potential and possible frameworks for addressing the issues. The second meeting reviewed the ultimate framework for GDP2 to vet core developmental capabilities that would be used in the pilot communities.

This framework was also reviewed through interviews with four experts on subjects relevant to our capabilities set and on measuring well-being more generally. These individuals had decades of experience studying and measuring the development of human capabilities. In this review, we asked these experts about missing capabilities and the need for capability refinement.

### Literature review

We conducted an exploratory literature review to identify and refine our capabilities. The team grounded its understanding of and starting list of capabilities in two conceptual frameworks: 1) the capabilities framework of Sen [8, 9] and Nussbaum [10], and 2) the seven principles of Life Course Health Development (health development, unfolding, complexity, thriving, timing, plasticity, and harmony) [11].

With these theories in mind, the team conducted a structured literature review, focusing on developmental descriptions of capabilities, using the PsychInfo, Education Resources Information Center (ERIC), and PubMed databases. For example, ‘engaging in community capability’ (described later) was searched using combinations of terms including community, civic engagement, environmental awareness, children, adolescents, and positive youth development.

The list of capabilities was iteratively refined using results from the literature reviews, TEP meetings, and key informant interviews. Once a final list of capabilities was developed, we conducted more extensive multidisciplinary literature reviews to characterize each capability and its ontogeny. These reviews identified the major domains contributing to each capability and the critical risk and protective factors for developing each capability during childhood and adolescence. The goal was to identify a) a justification for each capability, b) how the capability may develop across the life course, and c) factors that may influence capability development. This article only briefly describes these findings, and a more detailed review can be provided on request.

### Community pilot of the framework

We shared the final capability set with stakeholder groups from three diverse communities to obtain their reactions to the capabilities, identify capabilities that may be missing, and finally hear how they might use a sentinel measure such as GDP2. Above all, we hoped to understand how community leaders interpret and might use either individual capabilities or the core set of developmental capabilities. Early insight at the community level would allow the team to test both interest and understanding of the potential value of GDP2 and serve as an essential counterpart to the national exploration. Further, end-use is a core goal of this work to ensure that GDP2 enhances and informs local decisions. In keeping with a lead user approach for idea generation and social innovation development, we chose communities with a track record of embracing new and innovative approaches to advancing social change [12].

As such, we engaged with three communities with a history of civic engagement around measures of child and community well-being: Long Beach, California; Pasadena, California; and San Antonio, Texas. The communities were selected to facilitate engagement on a big idea, such as GDP2, given its potential complexity and ensure that community perspectives were grounded in exploring complementary concepts.

Communities were presented the broad descriptions of the capabilities to develop community-informed definitions of capabilities, learn which capabilities were considered priorities by communities, and help us understand what potential indicators we might seek to measure.

Two focus groups in each of the three cities were convened between August and October 2019. We invited participants representing diverse community sectors, including business, child welfare, civic groups, education, elected government, health, housing, parks and recreation, safety and justice, social/human services, and workforce sectors. Each focus group used small- and large-group discussions to elicit structured feedback. Participants were provided with an overview of the GDP2 framework and then engaged around specific capabilities. In groups of 2–3 persons, participants reviewed descriptions for specific capabilities. They wrote out ideas on poster boards for sub-capabilities, which are the skills or characteristics children or youths must develop to acquire a given capability. We also asked participants what might be missing from the capabilities list.

A research team member analyzed the qualitative data (i.e., participant-written poster notes supplemented with researcher notes from large-group discussions for clarification) in a spreadsheet to code for recurring themes across focus groups concerning each capability. We identified the sub-capability ideas mentioned in multiple focus groups from this process.

## Results

Our analysis generated seven developmental capabilities. We describe each below and summarize their key sub-capabilities (see Table 1). We also summarize some of the most salient considerations that emerged in the community pilot.

**Table 1:** Community respondent consensus around necessary sub-capabilities

Capability	Key Sub-Capabilities	Exemplar Phrases
Health	Knowledge to understand factors affecting health	*curriculum to know health/nutrition, understand the definition of health and linkage of sleep, activity, nutrition (The Performance Triad), able to access reliable information, make knowledgeable choices (discern), and find information re: what contributes to physical and mental health*
Needs	Develop the ability to advocate for self	*advocate for self, self-advocacy, and advocate (and actualize) for need fulfillment*
	Understand the interconnectedness of systems and resources for satisfying basic needs	*understand how systems affect how/whether needs are met, understand connections between education and economic opportunity, understand the value of education, and parent/caregiver intentionality of home, school, and community environment*
Communication	Develop empathy and emotional intelligence	*empathy – inherent worth of every human being, understand her emotions and has positive ways to express them (whether positive or negative), ability to name feelings and sit with it/step back, emotional regulation, recognize how feelings and emotions are connected to life/cultural experiences, and ability to self-regulate*
	Ability to navigate social media and technology use (e.g., non-verbal communication, trusted information)	*read non-verbal cues, the impact of non-verbal communication, navigate social media appropriately, the effect of technology on the ability to communicate, and skills to have appropriate management on those platforms*
	Be open to diverse people and communication	*assess communication styles (self, others), value diverse forms of communication, appreciate differences in temperament and POV, overcome language barriers, and let go of judgement*
Lifelong Learning	Be open to new and diverse viewpoints and experiences	*accept diversity of thought, reserve judgement and be available to LISTEN to new ideas/practices, needs to be intentional about exposure to different cultures, experiences, and viewpoints, courage to try new things, understand differences, value his perspective as well as others, learning and incorporating new knowledge, and take risks in trying new activities, methods, experiences*
	Ability to work collaboratively	*ability to do design thinking, crowdsource ideas, learn individually and collaboratively, work with others/connect with others, develop social skills, and work with others in collaborative and constructive ways*
Adaptation	Develop resiliency and coping skills	*build resiliency, develop those resiliencies and coping strategies to identify, understand, and deal with effects of stress, especially toxic stress, develop flexible resiliency and coping skills, accept change as own, be best in it, mindfulness/coping skills, and develop coping skills to manage change – and see its value for her growth; navigate crisis*
	Learn how to seek and access support	*destigmatize seeking help or resources, access immediate care/services/support to address challenges being faced, be proactive, and identify needs and reach out for necessary resources*
Connections	Develop empathy and emotional intelligence	*experience/learn empathy and compassion, be empathetic and exhibit care toward others and the world, empathy (better tolerance)/understanding others, let things go, release resentment, emotional intelligence, ability to self-regulate/soothe, and ability to communicate feelings and expectations*
	Ability to understand and appreciate diverse viewpoints and contexts	*experience/learn an appreciation for the global community, examine the facets of her identity in relation to the identity of other people, comfortably able to navigate within diverse contexts, see issues/situations from multiple perspectives, understand the difference as an asset/strength, ability to acknowledge bias, knowledge of others’ cultural and historical histories, personality differences, able to recognize individual and cultural differences as a positive value*
	Develop strong interpersonal skills and relationships	*needs to have emotionally skilled caregivers in early life – attachment, experience/learn unconditional love/intimacy and acceptance, value human relationships, better interpersonal skills/ability to recognize a cultural broker, feel connected, trust others, comfort with building relationships, and build social connection skills*
	Develop conflict resolution skills	*conflict resolution skills, navigate conflict in productive ways, able to forgive, and work through conflict with individuals and in groups*
	Develop self-reflection and self-awareness skills	*self-reflection and self-evaluation, self-awareness – understanding when you should move on together or apart, hold herself accountable, recognize fragile parts of her personhood, and adapt*
	Understand they are part of a larger system or social contract and its role in providing support or safety	*social contract – responsibility to each other, be part of a support system for self and others, feel safe and supported, understand how systems and institutions play a role in life, and see the external world as safe*
Community	Develop self-efficacy skills and empowerment	*hope/belief that there can be changes/make a difference, identify her agency and the power of individuals and groups within civic systems, ability to exercise power and build up others, she has a voice – and it matters, ability to access ‘power’, advocate, empowerment, and understand/believe her voice is valuable*
	Learn to understand the bigger picture of the community environment	*skills to understand the bigger picture, appreciate the interdependence within a community and its systems, ability to discern what ‘community’ means to them, understand systems, connected to the environment (everything around us, where we live, work, play), geographic connections, and understanding of how environmental services work at an early age and participate in their ‘environment’ and community*
	Ability to understand and appreciate diverse viewpoints and contexts	*effectively communicate divergent perspectives, understand a community situation/challenge from multiple perspectives, focus on assets, accept diversity, and value opinions of others – even if others disagree*
	Develop self-esteem and confidence to speak out	*possess positive self-esteem and confidence to speak truth to power, confidence to engage in discourse, feel safe and comfortable enough to be a voice, self-confidence, and strong sense of self – as an individual and part of a larger group*
	Understand and use social networks	*critically analyze social networks, connections to neighbors/social capital, help others see connections, parents/caregivers/community and/or mentors that encourage civic engagement, build trust, and understands value and importance of collective work (impact) – build coalitions*
	Develop a sense of responsibility and accountability	*knowledge of how values and norms constitute communities, accept responsibility, and become a contributor to their community*

Respondents identified listed sub-capabilities in two-thirds or more of the focus groups for each capability. Exemplar phrases are drawn from verbatim notes that respondents wrote during focus groups to describe this capability

### Developmental capabilities

#### Health: living healthy lives

Health capability is the ability to live a long and healthy life free from unnecessary suffering. It is foundational for all other capabilities. The Health capability includes the positive and negative dimensions of health and the development of physical and mental capabilities necessary to function in diverse settings optimally. This would also include the ability to adequately manage chronic health conditions and have access to high quality mental health resources. Living a long and healthy life is a component of human dignity [13].

Community leaders referenced two important considerations of this capability. First, having the Health capability does not necessarily mean that a person will realize a long, healthy life because that capability refers to an ability. Still, the outcome of longevity depends on personal choices and environmental exposures. A person may be highly capable but engage in unhealthy behaviors (e.g., smoking) or find him or herself in a situation, such as war, that creates environmental barriers to good health. Second, access to healthcare is an essential resource for developing the Health capability; however, it is insufficient for its full development. The skills needed for self-management to achieve health mature throughout childhood, and the complete acquisition of these skills is a hallmark of adult independence.

#### Needs: satisfying basic human requirements

The Needs capability is the ability to satisfy the requirements of nourishment, shelter, clothing, safety, play, sexuality, and physical activity. These needs are central to prevailing notions of human rights. Satisfying these needs depends on whether there are supportive, available, and interacting parental, familial, and community capabilities.

A consideration that emerged in community discussion is that to access these resources, an individual must be able to advocate for such access. Participants noted how little investment is provided to help people build essential advocacy skills.

#### Communication: expressing and understanding thoughts and feelings

The Communication capability is the ability of individuals to express themselves and understand others through verbal and non-verbal communication of thoughts and feelings. Communication is characterized by the acquisition of receptive and expressive language skills, the expression and regulation of positive and negative emotions, the ability to convey compassion, empathy, and gratitude, and the ability to read and appropriately react to one’s mental states and those of others (i.e., theory of mind).

Community leaders noted important 21st-century considerations. First, emotional regulation will be even more critical for managing one’s feelings in the face of challenges and stress. Second, communication skills are needed to combat increasing loneliness [14]. Many workplaces now demand not just cognitive skills but also emotional abilities to read, assess, interpret, and manage complex communication contexts effectively.

#### Learning: lifelong learning

The Learning capability is the ability of a person to learn across the life course as an individual and in groups and apply those learnings to new challenges, both inside and outside of formal education systems. In an information-based economy, not only are productivity increases based on learning, but they also are linked to improvements in health, adaptability, social cohesion, and other capacities associated with individual and social well-being [15]. Because complex developmental ecosystems “learn their way forward,” creating and advancing a learning society becomes a principal object of social innovation and improvement and a vital component of an agile and responsive policy framework.

Community leaders noted that lifelong learning is increasingly essential to expand knowledge or skills. The skills learned early in the life course during formal education can become rapidly outdated in contemporary workplaces [16]. The leaders noted that this capability also refers to abilities that enable individuals to participate effectively in group learning to obtain knowledge and solve problems, including efforts that employ combinations of human and machine intelligence [17].

#### Adaptation: adapting to change

The Adaptation capability is the ability of a person to adapt to life’s challenges, self-regulate behavior, and agilely adjust to rapid social, cultural, and technological change. Children and youth must overcome stress from diverse sources of adversity in situations of increasing uncertainty [18]. When children and youth reach the limits of their abilities to adapt, we witness downward spirals of disease, dysfunction, and loss of well-being.

Community leaders were keen on the adaptation capability. Technological and social change is accelerating, requiring children and youth to adapt. These challenges create mismatches between evolutionarily determined biobehavioral capacities and the demands of the modern world, which needs children and youth to adapt rapidly and efficiently to novel conditions and demands. Prioritizing adaptive capabilities can ensure that young people develop their abilities to preserve mental balance and flexibility in changing contexts [19].

#### Connections: connecting with others

The Connection capability is the ability of a person to develop and grow relationships with family, friends, intimate partners, and the natural environment. It refers to friendships and family relationships and the capacity for intimacy and love and our relationships with other species and the planet [20, 21].

Development of the ability to form relationships with others is enhanced by early cognitive, social, and self-regulatory health assets and reliable and responsive parental behaviors that elicit secure attachment, especially during infancy and early life [22]. Moreover, social connectedness contributes to well-being and acts as a buffer during times of stress [23].

Community leaders across all groups noted that developing connections with others required the capacity to be open to learning about and respecting diverse people and contexts. They also highlighted the importance of having positive interpersonal relationships to develop the skills needed to connect with others meaningfully. Community leaders also underscored the need to be able to see oneself as part of a more extensive social system and reference the social contract that makes explicit our responsibilities to each other.

#### Community: engaging in community

The Community capability is the ability to participate as an equal in a democratic society and make political choices that govern community life. In a world where individuals spend more time in virtual spaces, an individual may encounter, join, or leave many communities. Community engagement requires knowledge- and skills-based political and non-political competencies, values, and motivations [24].

Community leaders underscored this capability, noting that individuals must understand that they are part of a larger social fabric and acknowledge their responsibility to address community problems. The leaders noted the widespread lack of this capability and expressed concern that it weakens the human potential.

## Discussion

We have proposed and presented a new capabilities-based framework for measuring human potential that cities, counties, states, and the nation can use to monitor social progress. Moving beyond the GDP’s exclusive focus on economic productivity, the GDP2 is an alternative and complementary measure that focuses on the inputs to the potential for lifelong health, development, and well-being. Drawing on the empirical literature on life course health development, the GDP2 framework is an initial offering and very much a work in progress that will require further refinement, testing, and development. By holistically measuring physical, mental, and social health, this notion of human potential considers not only basic needs like food and housing quality and essential inputs like education, employment, and income but also factors of social and emotional development, a sense of purpose, social connections, and belonging.

Traditional economic and social progress measures like the GDP are not well suited for assessing the significant changes underway, nor does GDP illuminate potential options and alternative paths. As the trends in inequality, mortality, and child health suggest, the healthy development of children can have a significant impact on adult health, morbidity, mortality, education, and employment success [25]. But as the world rapidly changes, the capabilities and opportunities that children will need to realize their full potential will also evolve. As the goalposts of social and economic success continue to shift, the measures of success in childhood need to be adjusted accordingly.

Through extensive literature review and expert input, we developed a set of seven capabilities that community leaders affirmed as important for human potential measurement. While we included the environment in the connection capability, there remains a question about whether a distinct sustainability capability is required.

Unanswered questions remain. First, we continue to investigate whether Meeting Basic Needs should be a separate capability because it is so central across the other capabilities and could be considered a crucial part of adaptation or the health capability. Additionally, a lifespan framework will need to distinguish the multiple influences, levels, and development stages of each capability’s development. While some skills and abilities are necessary just to survive, there may be additional skills that enable someone to potentially thrive. We continue to ask how to incorporate equity in the capability framework appropriately. Finally, the new GDP2 measure must accurately represent the development of the seven capabilities and do it in a useful way to communities and the wide range of place-based health improvement initiatives.

Perhaps the biggest challenge to the use of the GDP2 framework is making it operational. How can we proceed to develop instruments that measure developmental capabilities? There are two primary options. One option is to create measures, de-novo, for each capability, test each measure's reliability and validity, and then consider combining the measures into an index or summary score. Creating seven capability measures and evaluating the usefulness and validity would be a resource-intensive and multi-year process. However, developing new measures of each capability allows us to capture precisely the data needed in the most parsimonious way.

The other option is to further develop the capabilities framework in collaboration with several communities and work with those communities to create a dashboard of existing indicators and indices. The measurement development and maturation process would proceed iteratively, based on community activation, innovation, and improvement efforts. The final product would include a portfolio of measures that could be adapted and customized as a GDP2 dashboard for any community.

Using existing measures and indicators to measure the seven capabilities has several strengths. It would save time and money and potentially generate an iterative learning process to speed innovation and measurement progress. Choosing from existing measures would allow selecting those with credibility with researchers, community stakeholders, and policymakers. Developing an indicator selection process with communities would also create an opportunity for greater community buy-in and launch a learning and measurement improvement process that could help build cross-sector camaraderie while at the same time moving beyond measurement toward collaborative action.

There are several possibilities for selecting indicators to populate an initial GDP2 dashboard for communities, cities, and states. One option is to map age-specific opportunity contexts by considering each capability’s risk and protective factors. This would provide important information about the underlying drivers of capability development using a life course development framework and would be actionable by communities by offering policymakers a specific intervention target. Creating an agile and adaptable GDP2 measurement system might have different features that allow communities to use existing data and choose which indicators to use based on their specific circumstances.

There are several limitations and considerations that future development of the GDP2 should address. This initial measurement approach focused on responding to conditions and needs in the United States, a high-income nation, with significant economic inequities, a strong emphasis individualism, and whose children have scored near the bottom on UNICEF league charts of child wellbeing for decades. While the developmental capabilities and process of potential refinement could be relevant to other high-income nations, additional testing and refinement would be necessary to globalize the GDP2 approach. Given the complexity of developing a new measure of human potential development, we began with experts and existing scientific literature and engaged with actively engaged “lead user” communities willing to participate in measurement development, recognizing that further work would require iterative co-design, adaptation, and learning. In addition to local testing and more end-user analysis and customization, comparative and experimental testing of different capability investment and enhancement strategies can help refine the measurement process and build momentum for adoption and use. Finally, this analysis did not incorporate a valuation of capabilities (both through market-based and non-market approaches), but this could be considered in future testing of the capabilities and identifying which capabilities would be prioritized.

## Conclusions

The radical uncertainties associated with today’s unprecedented changes pose fundamental questions about what it means to be healthy and what it takes to flourish. We need to rethink how society can help children and youth adapt in productive ways for a more promising future. Community leaders indicated that the GDP2 could elevate the importance and attention paid to positive health development pathways, which are leading indicators of future economic and social progress. The work aims to help families, researchers, providers, and policymakers reconsider what is possible in cultivating human potential and how more ambitious and far-reaching goals can be achieved. It provides a robust way to consider community and national investments in positive health and well-being.

## Data Availability

Data sharing is not applicable to this article as no datasets were generated or analyzed during the current study.

## Abbreviations

COVID-19: Coronavirus disease 2019
GDP: Gross Domestic Product
GDP2: Gross Developmental Potenital
SARS-COV-2: Severe acute respiratory syndrome coronavirus 2
TEP: Technical expert panel
